# Editorial: Deep learning for computer vision and measurement systems

**DOI:** 10.3389/frai.2026.1930978

**Published:** 2026-08-25

**Authors:** Seyed Jalaleddin Mousavirad, Mohammed El-Abd, Diego Oliva

**Affiliations:** 1 Department of Computer and Electrical Engineering, Mid Sweden University, Sundsvall, Sweden; 2 College of Engineering and Applied Sciences, American University of Kuwait, Salmiya, Kuwait; 3 Depto. de Ingeniería Electrónica - Fotónica, Universidad de Guadalajara, Guadalajara, Mexico

**Keywords:** computer vision, deep learning, measurement, medical measurement, pattern recognition

Deep learning has become a central technology in modern computer vision and measurement systems. By learning hierarchical and task-specific representations from data, deep neural networks have advanced image classification, object detection, segmentation, reconstruction, enhancement, and visual understanding. These developments have also influenced measurement systems, where accurate visual interpretation is essential for inspection, monitoring, diagnosis, localization, calibration, and decision support. Applications in manufacturing, robotics, healthcare, transportation, astronomy, cultural heritage, and autonomous technologies increasingly require systems that are not only accurate, but also robust, efficient and adaptable to real-world variability.

The aim of this Research Topic, *Deep Learning for Computer Vision and Measurement Systems*, is to explore how deep learning can enhance both computer vision methods and measurement-oriented systems. Traditional visual measurement approaches often rely on handcrafted features, controlled acquisition conditions, and fixed processing pipelines. Although these methods remain valuable, they can be limited by noise, illumination changes, occlusion, domain shift, limited annotated data and complex object structures. Deep learning methods, including convolutional neural networks, residual networks, encoder–decoder architectures, real-time detectors, and foundation models, have provided new opportunities to address these challenges (Goodfellow et al., 2016; He et al., 2016; Ronneberger et al., 2015; Redmon et al., 2016; Kirillov et al., 2023). The articles collected in this Topic demonstrate how these advances can be adapted to practical measurement scenarios across a wide range of domains.

A major theme of the Research Topic is visual inspection and structural assessment. Kumar et al. investigated automated damage diagnostics in fiber metal laminates using Mask R-CNN and the Segment Anything Model, demonstrating how deep learning can support non-destructive evaluation and quality assurance in advanced materials. In a related direction, Anusha and Anbarasi proposed a hybrid Swin Transformer with an enhanced feature representation block for crack detection in structural images. Their study highlights the potential of transformer-based models to capture both local spatial features and broader contextual information for infrastructure monitoring. Extending the role of visual measurement to urban management, Tanwer et al. evaluated vision transformers for detecting the fullness of garbage bins, linking computer vision with smart-city systems and more efficient waste collection.

Several contributions focus on difficult visual environments where measurement systems must operate under degraded, ambiguous, or low-contrast conditions. Zhang C. et al. introduced FSD-Net for underwater object detection, combining frequency- and spatial-domain feature enhancement to improve detection under blur, turbidity, noise, and poor visibility. Zhang J. et al. proposed a Laplace-guided fusion network for camouflaged object detection, showing that frequency-domain boundary information can help distinguish concealed objects from visually similar backgrounds. These studies illustrate an important trend: robust vision-based measurement often requires the integration of semantic information with fine spatial detail, edge awareness and domain-specific feature enhancement.

The Research Topic also addresses trust, authenticity, and safety-critical analysis. Uliyan et al. developed a video forgery detection method based on deep learning and multi-statistical features extracted from spatial and compression domains. This contribution is particularly relevant to visual forensics, where measurement systems must evaluate not only what is visible in an image or video, but also whether the visual evidence is authentic. In healthcare, Moustapha et al. presented a hybrid deep transfer learning framework for COVID-19 classification from X-ray images, combining pre-trained models, k-fold validation, and genetic-algorithm-based hyperparameter optimisation. Their work reflects the continuing importance of robust validation and model optimisation in medical imaging applications.

Other articles broaden the scope of deep learning for visual measurement into astronomy, cultural heritage, autonomous perception and vision-language learning. Al-Rajab et al. explored AI-based lunar phase detection by integrating lunar imagery with astronomical data, demonstrating how learning-based models can support scientific observation and the identification of the new crescent. Guan et al. proposed InfoMSD, an information-maximization self-distillation framework for parameter-efficient fine-tuning on artwork images, addressing the adaptation of large vision-language models to cultural heritage imagery. Du et al. introduced a mixture-of-prompts learning method for vision-language models, improving few-shot learning and generalization through prompt specialization and routing. Finally, Mane et al. presented IndiaScene365, a transfer learning dataset for Indian scene understanding under diverse weather and traffic conditions, emphasizing the need for representative datasets in unstructured and domain-specific environments.

Taken together, these contributions show that deep learning is moving computer vision and measurement systems toward more integrated, adaptive and domain-aware solutions. First, visual measurement is no longer limited to simple detection or classification; it increasingly involves segmentation, characterization, reconstruction, authenticity assessment, and decision support. Second, transformer architectures, vision-language models, and foundation-model-inspired approaches are becoming important tools for addressing limited data, complex scenes and domain shifts. Third, feature fusion, frequency-domain processing, transfer learning, self-distillation, synthetic data, and parameter-efficient fine-tuning are emerging as practical strategies for improving robustness and deployment feasibility.

Despite these advances, several challenges remain. Measurement-oriented deep learning systems must be evaluated not only by accuracy, but also by uncertainty, repeatability, calibration stability, interpretability, computational efficiency, and reliability under changing operating conditions. Future research should place greater emphasis on trustworthy and explainable AI, standardized validation protocols, lightweight models, multi-sensor fusion, self-supervised learning, and generalization across domains. Collaboration between computer vision researchers, measurement scientists, engineers, clinicians, industrial practitioners, and domain experts will be essential for translating algorithmic progress into dependable real-world systems.

We thank all authors, reviewers and editorial staff for their valuable contributions to this Research Topic. We hope that this Research Topic will stimulate further research on robust, interpretable and efficient deep learning methods for computer vision and measurement systems, and will encourage new applications across industry, healthcare, robotics, environmental monitoring, scientific imaging, cultural heritage, and autonomous technologies.
